# 

**Published:** 1915-08-21

**Authors:** 


### EX-MAYOR'S GIFT TO BURNLEY HOSPITALS.

Mr. Thomas Thornber, of Burnley, Lancs., cotton spinner and manufacturer, for four years Mayor of Burnley, who left unsettled property of the gross value of £148,646, has bequeathed £10,000 to his trustees for distribution for charitable purposes within the borough of Burnley; and according to a recently issued statement this amount has been allocated as to £5,000 to the Burnley Victoria Hospital, £1,000 to the Burnley District Nursing Association, and various sums to other charities of a religious and social character.

### Buildings Contemplated and in Progress.

Bourne.—The Kesteven County Council has decided to erect a tuberculosis block in connection with the Bourne Infectious Diseases Hospital. The cost is estimated at £100.

Brixham.—The Urban District Council has agreed to purchase a site for an isolation hospital at a cost of £300.

Edmonton.—Tenders are being invited by the Guardians of Edmonton for the erection of two temporary buildings for orderlies at the Edmonton Military Hospital in accordance with plans and specifications prepared by the architect, Mr. J. C. S. Mummery, of 13 Fitzroy Square, W., from whom full particulars can be obtained.

Hambledon.—The Rural District Council is inviting offers for the purchase of their temporary hospital at Hankley Common, in the parish of Elstead. The buildings consist of a ward block, a lodge, and cart shed, and could be easily removed. Full particulars are to be obtained from the Council's surveyor, Mr. E. L. Lunn, of High Street, Guildford.

Holsworthy.—With a view to effecting joint action in the provision of a small-pox hospital, the Rural District Council proposes to approach the Holsworthy Urban District Council.

Houghton Green.—The Warrington Corporation propose to erect a small-pox hospital between Orford and Houghton Green, estimated to cost £1,500.

St. Anne's.—Plans have been passed by the Urban District Council for a military convalescent hospital on the Clifton Park Racecourse for the Blackpool Corporation.

Sleaford.—A sub-committee of the Urban District Council has been appointed to confer with the Rural District Council upon the question of erecting a joint isolation hospital.

Teignmouth.—A joint scheme for the provision of small-pox accommodation is under consideration by the Urban District Councils of Teignmouth and Dawlish.

Weston.—According to a communication received recently by the Mayor of Bath, the War Office has decided to build a military hospital on the Lansdown Cricket-ground at Coombe Park, Weston.

### Tenders.

### Metropolitan Asylums Board.

Park Hospital.—A tender by A. H. Inns, 36 Camomile Street, E.C., amounting to £116, for painting work at this hospital, has been accepted by the Board.

Northern Hospital.—A tender by the same firm, amounting to £123, for cleaning and repairs at this hospital, has been accepted.

Queen Mary's Hospital.—A tender by W. Hussey, of 32 Albert Hall Mansions, S.W., amounting to £102, for roof repairs and painting at this hospital, has been accepted.

North-Western Hospital.—A tender by W. H. Wheeler and Co., Ltd., 14 New Kent Road, S.E., amounting to £94, for tar macadam work and tar paving work, has been accepted.

### Gifts and Bequests.

Mrs. Hamilton-Fellows has generously defrayed the cost of the erection of an outdoor shelter for the patients in the London Lock Hospital's grounds.

Mr. James Percy, aged eighty-one, of Ealing Common, steamship owner, has left £100 to the Ealing Cottage Hospital and Dispensary, and £100 to the West London Hospital, Hammersmith.

A donation of £10 has been received by Addenbrooke's Hospital, Cambridge, from "Anonymous," Ipswich, in recognition of treatment received many years ago—a practical expression of gratitude.

Mrs. Susan Jane Malmberg, of Torquay, has left £500 each to the Anti-Vivisection Hospital, Battersea, and the Mission to Lepers; and £200 each to the Society for the Prevention of Cruelty to Animals and the London and Provincial Anti-Vivisection Society.

Mr. John Stevenson Kershaw, of Littleborough, flannel manufacturer, who left estate valued at £166,472 gross, has bequeathed £2,000 each to the Rochdale Infirmary and the Manchester Infirmary, £1,000 each to Henshaw's Blind Asylum, the Rochdale District Nursing Association, and the Rochdale Deaf and Dumb Institution, and £500 to the Rochdale and District Society for Visiting and Instructing the Blind.

The Leicester Royal Infirmary has received, or been notified of, legacies from the following: Mr. J. H. Burley, £5,000 to the infirmary and £2,000 to the Children's Hospital; Mr. M. H. Saulsbury and Mr. Alfred Green, £500 each to the Children's Hospital; Mr. S. F. Stone, £250; Mrs. E. L. Pochin, £200; Mr. G. G. Johnson, £100; Miss E. K. Varnam, £100; Miss S. N. Beasley, £89 10s.; Mrs. Preston, £50; Mr. Alfred Cooper, £20.

The Committee of Addenbrooke's Hospital, Cambridge, have received notification of the following bequests: Mrs. Edwards, of Impington, £100; Miss Sophia Kent, of Wilbraham, £200, duty free; Alderman George Kell, J.P., of Cambridge, £100, duty free. The late Alderman Kell was for many years a governor and a member both of the old weekly board and the General Committee and took an active interest in the hospital. Mrs. Louisa Augusta Murray Gartshore, £100, in memory of her father, the Rev. W. K. Douglas, fellow and tutor of Trinity College, Cambridge. In the case of benefactors of £100 the executor of the will, or if there be more than one, then one of them duly nominated may be appointed an honorary governor for life in pursuance of the bye-laws.

### ELECTRICAL TREATMENT AT MAIDA VALE HOSPITAL.

The next course of instruction in Electrical Treatment at the Hospital for Epilepsy and Paralysis, Maida Vale, commences on September 3, and the application for admission should be addressed to the Secretary.

### ARUNDEL CASTLE.

The Duke of Norfolk has arranged for Arundel Castle and grounds, with the keep, dairy, and Fitzalan Chapel, to be open to visitors on payment, on Tuesday afternoons, from September 21.

The receipts will be divided between Sussex hospitals, including the Arundel Emergency Hospital, and the Red Cross Society.

### Latest Installations in Hospital Equipment.

### WHAT OTHERS ARE DOING.

***** We desire information calculated to keep this column up to date in the interest of institutional workers generally. Secretaries and other executive officers are invited to forward particulars of new installations in any department. In this way a file will be available for all hospital authorities contemplating improvements and anxious to know what other institutions have found effective and modern.**

The Red Cross Hospitals at Trafford Hall, Hollymoor, Birmingham, and Werneth have installed potato-peeling machines, No. 3 "Rapid" and No. 3 "Premier" respectively, manufactured by Mabbott and Co., Phœnix Iron Works, Poland Street, Manchester.

The 2nd Northern General Hospital, Priestley Hall, Leeds, has put in a new potato-peeling machine designed and manufactured by Messrs. Mabbott and Co., Phœnix Iron Works, Poland Street, Manchester.

The Glasgow New Royal Hospital for Sick Children, where accommodation for wounded soldiers has also been provided, has recently been installed with 100 Nesbit-Evans balcony bedsteads of the adult size for the treatment of wounded soldiers. At the Glasgow Military Hospital at Cambuslang 250 Nesbit-Evans bedsteads are in use, and the Scottish Branch of the British Red Cross Society has obtained 1,000 Nesbit-Evans bedsteads for their Emergency Hospitals at Woodside and Bellahouston, near Glasgow. The firm's address is J. Nesbit-Evans and Co., Floodgate Street, Birmingham.

The St. John Ambulance Brigade have installed in their hospital at Etaples an electric-lighting installation, comprising upwards of 700 lights, and also fire-alarms and telephone installations. The work has been carried out by Messrs. H. J. Cash and Co., Ltd., of Caxton House, Westminster, London, S.W.

The Liverpool merchants have installed at their mobile hospital at Etaples an electrical installation, comprising upwards of 250 lights. The work has been carried out by Messrs. H. J. Cash and Co., Ltd., of Caxton House, Westminster, London, S.W.

### The Book Market.

LIST OF NEW BOOKS RECEIVED FROM THE FOLLOWING PUBLISHERS:—

H. K. Lewis and Co., Ltd.: "Health in the Camp: A Talk to Soldiers." By H. R. Kenwood. 3d. net.

Oxford University Press: "Infant Health: A Manual for District Visitors, Nurses, and Mothers." By J. S. C. Macmillan. 2s. net.

University of London Press: "Germany's Food: Can It Last?" English Version. Edited by S. Russell Wells, M.D. 2s. net.

### Pamphlets and Papers.

Grand United Order of Odd Fellows: Monthly Magazine. August.

The Optimist. Being the Camberwell House Magazine. July 1915. Price 6d.

An Empire Without Taxes and, practically, Without Rates: How to abolish them and at the same time to pay for the war; to pay off the National Debt, etc., without expense to anyone. By John Kyte Collett, Cardiff. Price 1d.

Rates of Subscription: Twelve months, 6/6; Six months, 4/-; Three months, 2/-, post free to any address in the United Kingdom. Abroad, Twelve months, 8/8; Six months, 5/-; Three months, 2/6, post free.—Address The Subscription Dept., "The Hospital," The Hospital Building, 28/29 Southampton Street, Strand, London, W.C.

